# Impact of Gravity on Thyroid Cells

**DOI:** 10.3390/ijms18050972

**Published:** 2017-05-04

**Authors:** Elisabetta Albi, Marcus Krüger, Ruth Hemmersbach, Andrea Lazzarini, Samuela Cataldi, Michela Codini, Tommaso Beccari, Francesco Saverio Ambesi-Impiombato, Francesco Curcio

**Affiliations:** 1 Department of Pharmaceutical Science, University of Perugia, San Costanzo, via Romana, 06121 Perugia, Italy; elisabetta.albi@unipg.it (E.A.); samuelacataldi@libero.it (S.C.); michela.codini@unipg.it (M.C.); tommaso.beccari@unipg.it (T.B.); 2 Clinic and Policlinic for Plastic, Aesthetic and Hand Surgery, Otto-von-Guericke-University, Leipziger Str. 44, 39120 Magdeburg, Germany; marcus.krueger@med.ovgu.de; 3 German Aerospace Center (DLR), Institute of Aerospace Medicine, Gravitational Biology, Linder Höhe, 51147 Cologne, Germany; ruth.hemmersbach@dlr.de; 4 Laboratory of Nuclear Lipid BioPathology, CRABiON, Perugia, via Ponchielli 4, 06073 Perugia, Italy; andrylazza@gmail.it; 5 Dipartimento di Area Medica (DAME), University of Udine, p.le M. Kolbe 4, 33100 Udine, Italy; ambesis@me.com

**Keywords:** thyroid gland, thyroid cancer, microgravity, hypergravity, space environment

## Abstract

Physical and mental health requires a correct functioning of the thyroid gland, which controls cardiovascular, musculoskeletal, nervous, and immune systems, and affects behavior and cognitive functions. Microgravity, as occurs during space missions, induces morphological and functional changes within the thyroid gland. Here, we review relevant experiments exposing cell cultures (normal and cancer thyroid cells) to simulated and real microgravity, as well as wild-type and transgenic mice to hypergravity and spaceflight conditions. Well-known mechanisms of damage are presented and new ones, such as changes of gene expression for extracellular matrix and cytoskeleton proteins, thyrocyte phenotype, sensitivity of thyrocytes to thyrotropin due to thyrotropin receptor modification, parafollicular cells and calcitonin production, sphingomyelin metabolism, and the expression and movement of cancer molecules from thyrocytes to colloids are highlighted. The identification of new mechanisms of thyroid injury is essential for the development of countermeasures, both on the ground and in space, against thyroid cancer. We also address the question whether normal and cancer cells show a different sensitivity concerning changes of environmental conditions.

## 1. Introduction

The effects of the space environment are mainly due to cosmic radiation, microgravity, and a confined habitat. Physical and mental equilibrium is very likely to be subject to significant perturbations during prolonged space missions. Astronauts are subjected to several physiological variations, such as cardio-circulatory, musculoskeletal, and immune disorders, together with changes of mental conditions, mood, and personality during and after re-entry from space missions [1]. The space research in the field is currently attracting more and more attention among researchers since, in the near future, an increasing number of astronauts will visit the International Space Station (ISS) and beyond for prolonged times and space is presently considered as the “next frontier” for humankind [1].

The mammalian thyroid gland consists of two lobes structurally composed by follicles and interfollicular spaces. Follicles are surrounded by thyrocytes which synthesize triiodothyronine (T3) and tetraiodothyronine (T4), while C cells in interfollicular spaces secrete calcitonin [2].

The diversity of cell types and the complexity of its hormone functions render the thyroid gland particularly relevant in the functioning of the entire organism. Therefore, maintaining good physical and mental health requires a correctly functioning thyroid gland.

## 2. How Gravity Influences Thyroid Function

All living organisms have evolved under the influence of the constant gravity force on Earth, which maintains the architecture and function of each kind of cell. The human organism possesses a series of adaptations to gravity changes not only at systemic, but also at the cellular level, such as the regulation of the circadian rhythm, the activation of mechanotransduction pathways, and inducing modifications in immune response, metabolism, and cell proliferation [3,4,5]. Additionally, genes that modify chromatin structure and methylation have been identified, suggesting that long-term adaptation to gravity may be mediated by epigenetic modifications [5].

### 2.1. Simulated Microgravity

Usually the impact of microgravity is simulated on the ground by different approaches: prolonged head-down tilt bed rest in humans induces comparable physiological changes with respect to fluid distribution, muscle, and bone loss as in space missions. Small animals, plants, and cell cultures are studied in ground-based facilities under conditions of a randomized influence of gravity. In the case that the exposed system does no longer perceive gravity as a stimulus, and the method applied does not induce non-gravitational effects, the term “simulated microgravity” is justified. Our assumption bears several prerequisites. The exposed system should possess a sensor for the detection of gravity and the threshold of the system should be less than the residual acceleration induced by the experimental conditions. In case of thyroid cells, a gravisensor has not yet been identified and, consequently, a threshold of graviperception is unknown. However, this type of cell reveals a large number of physiological changes under altered gravity conditions which might bring us closer to the identification of a general cellular gravisensory mechanism, which has also been postulated for other cell systems [6].

Microgravity is characterized by the prevention of sedimentation. On the ground this is achieved on so-called clinostats, in which samples are rotated around one axis positioned perpendicular to the direction of the gravity vector. By placing the sample container in the center of rotation and keeping its diameter small (in the range of a few mm) the induced residual accelerations can be kept as minimal as possible. A clinostat with one rotation axis (2D clinostat) is operated constantly in one direction which results in a static change of the gravity vector and in turn—at appropriate speed—prevention of sedimentation.

3D clinostats and random positioning machines (RPM) are further machines used for ground-based microgravity experiments aiming to simulate microgravity conditions [7,8]. Here, two rotation axes are mounted in a gimbal mount and their movement with respect to speed and direction is controlled by an algorithm [9]. While 3D clinostats are commonly rotating continuously, but with changed speed of the two axes at random, in a random positioning machine not only the velocity, but also the direction of rotation is altered in a real random mode. Comparative studies between the different experimental approaches are necessary to identify, experimentally, the induced non-gravitational effects, such as shear forces [8,10] and to avoid misinterpretations. Finally, simulations have to be verified in real microgravity.

**Figure 1:**
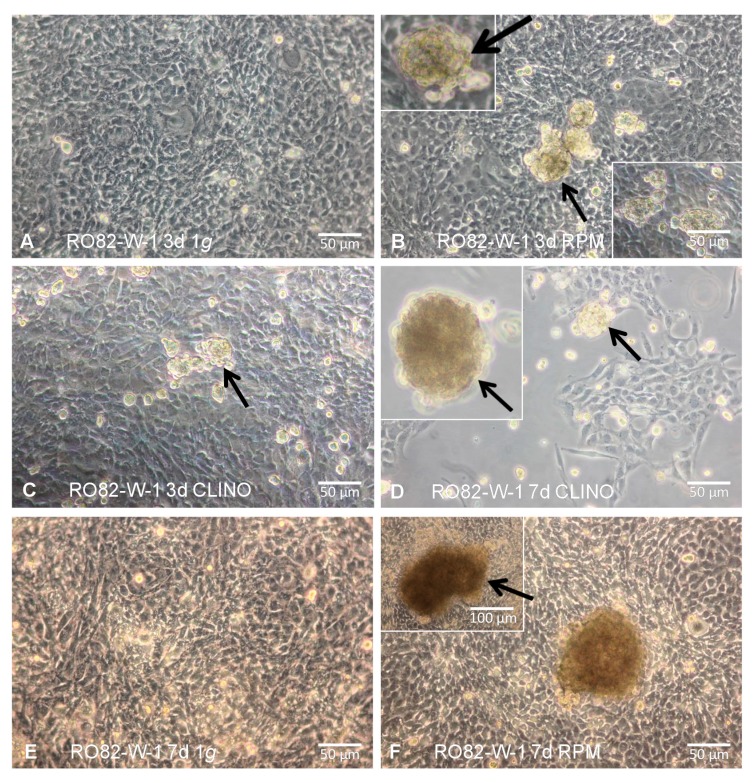
Formation of 3D spheroids: RO82-W-1 cells cultured for 3/7 days at static 1× *g* results in a 2D monolayer, while incubation on the RPM or on a clinostat shows 3D aggregates (multicellular spheroids; MCS). (**A**) RO82-W-1 cells cultured for 3 d at static 1× *g*; (**B**) MCS (arrows) formed on the RPM after 3 days; (**C**) MCS (arrow) formed on the clinostat after 3 days; (**D**) MCS (arrows) of RO82-W-1 cells cultured for 7 days on the clinostat; (**E**) RO82-W-1 cells cultured for 7 days at static 1× *g*; (**F**) Adherent cells and large MCS (arrow) formed on the RPM after 7 days. For more details see [17].

Normal thyroid cells (HTU-5 strain) cultured on a 3D clinostat (with a rotation of 60°/s) show apoptotic signs highlighted by electron microscopy analysis, activation of caspase-3, increase in Fas and Bax, and elevation of 85-kDa apoptosis-related cleavage fragments resulting from enhanced poly (ADP-ribose) polymerase activity [11]. Within 12 h of 3D clinorotation the monolayer of human follicular thyroid carcinoma cells (ML-1 line) turns spontaneously into three-dimensional multicellular tumor spheroids with an increase of extracellular matrix proteins and of TGF-β1, while thyroglobulin, fT3 and fT4 secretion are reduced [12] (Figure 1). Within 24–48 h of exposure on a random positioning machine (RPM), ML-1 cells show an upregulation of intermediate filaments, cell adhesion molecules (vimentin and vinculin), extracellular matrix proteins (collagen I and III, laminin, fibronectin, chondroitin sulfate), and Fas protein, while Bcl-2 is downregulated [13]. Laser scanning confocal microscopy of HTU-5 and ML-1 cells immuno-stained with anti-cytokeratin demonstrate that cytokeratin filaments extend from the center, are thickened, coalesce, and shortened, while the vimentin network forms a coiled aggregate, more closely associated with the nucleus as compared to control cells [14]. Changes of enzymes involved in carbohydrate metabolism, protein synthesis, and degradation are present in HTU-5 normal thyroid cells, in FTC-133, and in CGTH W-1 thyroid cancer cells [15]. During long-term exposure on a RPM (7–14 days) thyroid cancer cells form larger and more spheroids than normal thyroid cells, what can be related to an earlier production of the cell adhesion molecule osteopontin [16].

### 2.2. Real Microgravity

Short-term effects of real microgravity—in the order of seconds—can be studied with research platforms such as drop towers or parabolic flights (during which microgravity lasts a few seconds), and by sounding rockets (microgravity condition lasts minutes). During the TEXUS-44 mission (launched on 7 February 2008 from Kiruna, in Northern Sweden), FRTL-5 cells were treated with TSH (Thyroid-stimulating hormone) at the onset of microgravity and were fixed after 6 min and 19 s, just at the end of the microgravity period [18]. The study has clearly shown that FRTL-5 cells undergo relevant changes in real microgravity conditions: they aggregate, chromatin condensates, and TSH receptors are shed in the culture medium. This caused impaired production of cAMP and of proteins involved in cell signaling, such as PKC-ζ, PPAR-γ, and SMase and, consequently, failed to respond to TSH stimulation [18]. Live-cell imaging experiments with the fluorescence microscope FLUMIAS during a parabolic flight campaign and the TEXUS-52 mission, showed cytoskeletal changes of FTC-133 cells in real-time. These changes occur rapidly after entrance into microgravity. Under the microscope disturbance of F-actin bundles were detected. Another important finding was the formation of filopodia- and lamellipodia-like structures [19].

### 2.3. Hypergravity

During the initial launch phase of parabolic flights and spaceflight, hypergravity forces due to rocket acceleration are accompanied by launch vibration. In longer space missions hypergravity is not such an important issue, however, during parabolic flights each of the approximately 30 parabolas normally include about 22 s of microgravity intercalated by periods of normal and hypergravity, which may influence structural and functional changes in plant, animal, and human cells. In ML-1 thyroid cancer cells hypergravity (1.8× *g*) does not change *ACTB*, *KRT80*, or *COL4A5* mRNA for extracellular matrix proteins, but upregulates the mRNAs for the metastasis suppressor protein 1 and for the cytoskeleton-associated LIM domain and actin binding protein 1 [20]. Experiments with Nthy-3-1-ori cells at 1.8× *g* showed that *ITGA10* expression is not gravity-dependent in normal thyroid cells [21]. Hypergravity is also studied on the ground by slow-rotating centrifuges [22]. The exposition of TSH-stimulated FRTL5 cells to hypergravity (5× *g* and 9× *g*) increases cAMP production [23]. In ML-1 cells in culture, centrifugation at 1.8× *g* induces significant variations of growth factor mRNAs. A PKCα-independent mechanism of *IL6* gene activation seems to be very sensitive to physical forces [24]. In rats, hypergravity increases TSH response and T3 production [25]. Repeated five-day 2× *g* treatment of rats influences cell structure, enzymes, such as SMase and SM-synthase, and hormone content in the thyroid gland, which differ in quantity or quality from the changes arisen under the primary five days at 2× *g*, pointing out the animal capability for “memorizing the change of gravity level” [26]. The 2× *g* exposure upregulates TSHR (thyroid stimulating hormone receptor) surface proteins in mouse thyroids, but the response to TSH treatment remains unchanged without variations of cAMP, because hypergravity delocalizes TSHR [27]. In fact, immunofluorescence analysis of TSHR demonstrates that in control mice maintained in the vivarium the receptor is present on the surface of thyrocytes that surrounded follicles with a precise location, whereas in 2× *g* samples the fluorescent signal is higher and spreads over the entire surface of the thyrocytes [27]. The cell membrane loses the β-subunit of the receptor in the culture medium together to cholesterol (CHO), whereas SM remains unchanged [28]. On the other hand, the SM metabolism also has no variations; in fact, SMase and SM-synthase 1 expression is not affected by 2× *g* exposure [29], indicating that membrane CHO, and not SM, is critical for TSH–TSHR interaction. In addition, 2× *g* exposure induces the loss of parafollicular cells and the reduction of calcitonin production [30].

## 3. Space Environment Drives Thyroid Cells in Culture toward the Change of Cell Phenotype

Proliferating and quiescent FRTL5 cells have been used in two different space missions performed by the European Space Agency (ESA): the Eneide mission with astronaut Roberto Vittori (15–25 April 2005), and the Esperia mission with astronaut Paolo Nespoli (23 October–7 November 2007). In these missions, the behavior of thyrocytes in culture in the space environment has been analyzed [30]. During space missions, FRTL5 cells do not respond to TSH treatment; the space environment influences cell proliferation causing a pro-apoptotic condition in cells: this status is characterized by specific expression levels of STAT3, RNA polymerase II, and Bax, and by Smase- and SM-synthase-specific activities [31]. The SMase/SM-synthase activity ratio is very high in apoptotic cells, lower in pro-apoptotic cells, low in proliferating cells, and very low in quiescent cells. A comparison of the SMase/SM-synthase ratio value in space mission cells with that of proliferating, quiescent, pro-apoptotic, and apoptotic cells demonstrates that the space mission cells are in a pro-apoptotic state; thus, the SMase/SM-synthase ratio has been proposed as a biomarker for thyroid cell fate very useful in space missions [31]. FTC-133 analysis at the re-entry from the Shenzhou-8 mission (November 2011, 10 days) shows that the space environment induces a scaffold-free formation of extraordinarily large three-dimensional aggregates [32] and changes in the expression of genes and proteins involved in cancer cell proliferation, metastasis, and survival, shifting the cells toward a less tumor-aggressive phenotype [33]. Strong evidences from lipidomics studies, cancer preclinical models, and clinical trials have shown the key role of lipid molecular species in supporting cancer generation and progression. Such effects may result from fundamental changes in lipid raft composition, persistent ER stress which, through three distinct ERS sensor proteins: ATF6 (activating transcription factor 6), PERK (protein kinase RNA-like endoplasmic reticulum kinase) and IRE1 (inositol-requiring trans-membrane kinase/endonuclease 1), activate the unfolded protein response (UPR), and disruption of the lipid-mediated crosstalk between cancer and stromal cells. All of the processes mentioned above may be influenced by the space environment.

### 3.1. Thyroid Glands of Wild-Type and Transgenic Mice on Board the International Space Station

The effect of long-term exposure to the space environment on thyroid glands in vivo was shown for the first time by participating in the longest-duration spaceflight mission ever endured by any living animal within the “tissue sharing” team headed by Cancedda [1]. During the mission, six mice had been exposed to the space environment for 91 days (28 August–27 November 2009) on board the International Space Station (ISS), while kept inside the “mouse drawer system” (MDS). Both wild-type (WT) and transgenic mice (TG) with an overexpression of the pleiotropin (PTN) gene, which is under the control of the same specific human bone promoter of the osteocalcin gene, have participated in the mission. The thyroids of TG mice were analyzed with respect to bone metabolism in space and in a corresponding 1× *g* reference on the ground. Post-flight thyroid glands showed changes in follicular and parafollicular cells with different modifications in signal lipid and protein content [1,28,30].

### 3.2. Structural Changes

No significant changes in the proportions of major and minor axes of the glands between mice in space or on the ground were noted, but in the thyroid gland of WT ground control follicles showed variable size and spatial orientation. In contrast, spaceflight animals have a more homogenous thyroid tissue structure, with ordered follicles in which thyrocytes are thicker and the nuclear volume is increased; consequently, the thyroid epithelium vs. colloid volumetric ratio is higher in space than on the ground [1]. In addition, the interfollicular space is strongly reduced with the loss of C cells and defects of calcitonin production [30]. Since, on the ground, a spatial integration of follicular and parafollicular cells, and a functional coordination of both epithelial cells, are observed [34], the space environment evidently induces modifications of follicular cells responsible for C cell changes, causing defective bone homeostasis via thyroid disequilibrium [30]. The loss of thyrocytes arranged in a continuous rim around the colloid and irregularity in the parafollicular spaces with the reduction of calcitonin expression may, in part, be due to the confinement in MDS [35]. Overexpression of PTN on the ground does not protect the thyroid gland from the confinement-induced calcitonin reduction [35], whereas the space environment counteracts the effect of confinement, retains the thyroid C cells in shape, and strongly reduces the loss of C cells, thus exerting a protective action [30].

### 3.3. Modulation Ligand-Receptor: TSH–TSHR

In WT mice, the space environment induces both TSHR increased expression and different cell distribution, in comparison with the control mice maintained in the vivarium [1]. While in control mice thyroids, the TSHR is distributed uniformly on the thyrocyte surface, after spaceflight the receptor localizes instead in the intracellular junctions and cell membranes, while it is not found in the nuclei. Additionally, caveolin-1 is overexpressed in the space environment and its distribution is highly consistent with the TSHR localization [1]. It has been demonstrated that TSHR is a G protein-coupled receptor [36] associated with both no-raft and raft fractions of cell membranes [37]. In cancer cells, a wide range of signaling proteins and receptors regulating pro-oncogenic and apoptotic pathways during all stages of carcinogenesis reside in lipid rafts. Importantly, lipid rafts and their main component, cholesterol, are increased in the membranes of many types of cancer cells, as well as in the membranes of tumor-released exosomes. Important oncogenetic pathways and their aberrant activation correlate with increased lipid rafts. Lipid rafts may also be involved in cancer dissemination: they participate in cancer cell migration by regulating cytoskeletal reorganization and focal adhesion functions. Cholesterol-depleting agents inhibit the formation and activation of specific lipid raft entities called “clusters of apoptotic signaling molecule-enriched rafts” (CASMERs), which are co-aggregations of lipid rafts with death receptors and their downstream apoptotic molecules. They activate the apoptotic response independently of death receptor ligands.

Furthermore, lipid rafts are rich in SM, CHO [38], and caveolin-1 [39]. SM present in lipid rafts is rapidly metabolized with SMase and SM-synthase enzymes by changing the structure/function of rafts and generating the lipid second messengers involved in signal transduction [40]. The space environment increases the levels of SMase and SM-synthase, moves SMase from the nucleus to the cytoplasm and the cell membrane, and increases its activity [29]. Therefore, in the space environment there is a remodeling of cell membranes which become rich in lipid rafts containing TSHR, caveolin-1, and SMase. This induces the thyroid gland to respond to TSH treatment more intensively as compared to the thyroids of control mice. Thus, the cAMP release in spaceflight animals is higher than that of control animals [1]. Thyrotropin regulates the TSHR-rafts complexes: TSH activates cells through a specific signal transduction pathway and causes the disappearance of the raft-TSHR complexes because it stimulates their monomerization and rapid exit [39]. The production of cAMP by activation of the TSHR, continues in the pre-Golgi compartment and the spatial patterns of downstream signals are influenced by the location of the TSHR-cAMP signal [41]. Cytosolic protein kinase A (PKA) I and PKA II, which are mainly located in the Golgi complex, are activated by cAMP that spreads across the basolateral membrane; several other targets, situated in different cellular compartments, are also phosphorylated by PKAs [42,43]. The increase of TSHR in the thyroids of spaceflight animals is a compensatory mechanism, and explains the strong response to TSH after stimulation. The stress is, in part, due to the confined environment since, in the thyroids of mice maintained in MDS on the ground, intermediate changes between those of control spaceflight animals are observed [35]. During the confinement, TSHR and cAMP slightly increase without any displacement of the receptor within the cell, while no changes were found in SMase expression, localization and activity [35]. Therefore, confinement can only be a predisposing factor and the data in spaceflight animals are due to the combination of stress, confined environment, microgravity, and radiation [35].

### 3.4. How Space Environment Affects Cancer Proteins in Thyroid Glands

In the thyroid gland, Galectin 3 (Gal-3) plays a significant role in the pathogenesis of well-differentiated carcinoma, particularly in papillary carcinoma [44]. Gal-3, together with human bone marrow endothelial cell-1 (HBME-1) and Cytokeratin-19 (CK-19), are markers most commonly used to assist in distinguishing different thyroid lesions [45]. In addition, MIB-1 index is useful for evaluating proliferative activity and predicting the aggressiveness of thyroid carcinoma [46]. In the space environment, the MIB-1 proliferative index and CK-19 are negative in the thyroid tissue, whereas HBME-1 and Gal-3 show a higher expression in comparison with thyroid tissue in 1× *g* controls [47]. Gal-3, usually present in the cytoplasm, nucleus, and extracellular space, diffuses from thyrocytes in the colloid because of the remodeling of cell membrane proteins and lipids which occurs in microgravity. Alshenawy suggests that no single marker is completely sensitive and specific for the diagnosis of thyroid lesions, but only their combination [48] and Gal-3 + HBME-1 is considered highly significant for distinguishing benign from malignant lesions [49]. The possibility that HBME-1 and Gal-3 overexpression might indicate a thyroid tissue premalignant state cannot be excluded, considering that, in microgravity, follicle cells appear two times larger with darker colloids [1], similar to those of papillary carcinoma [50].

## 4. Cancer versus Non-Cancer Cells

When reviewing literature, the question becomes obvious whether normal cells differ in their sensitivity towards a change in the influence of gravity compared to cancer cells. Furthermore, we have to answer whether spaceflight induces carcinogenesis, understand the underlying mechanisms, and develop countermeasure or protection devices. Studies with normal and cancer thyroid cells might bring us closer to the answers. Table 1 reviews the current data.

Table 1 shows that normal, as well as cancerous, thyroid cells react to altered gravity conditions. In addition to morphological changes (the induction of spheroid formation), primarily, the gene expression of those genes, which are involved in cytoskeleton formation, cytoskeleton modulation, and extracellular matrix, is altered. Up to now, it is difficult to say whether normal cells are more sensitive than cancer cells. Ivanova et al. found that long-term exposure to hypergravity stimulated cGMP efflux in cultured human melanocytes and non-metastatic melanoma cells, whereas highly-metastatic melanoma cells appeared to be insensitive to hypergravity, most probably due to an upregulated cGMP efflux at 1× *g* [59].

At the current status, a statement of a differential gravity response of normal versus cancerous thyroid cells is difficult. It cannot be excluded that experimental parameters, for example, radiation, hardware geometry, cell density, and experiment operations during spaceflight as well as in ground facilities influence the results. Thus, they have to be clearly defined and described. Further experiments are needed to address the gravity impact on thyroid function with respect to the risk of astronauts to develop thyroid cancer during long-term spaceflights.

## 5. Summary and Perspective

Life, as we know it in our planet, evolved not taking into account the effects of microgravity and space radiation. The space environment induces several changes in thyroid glands under the influence of the pituitary gland. These observations may be instrumental for developing protective measures and countermeasures, to be adopted for the health and safety of all individuals exposed on Earth to extreme living and/or working conditions, and to astronauts prior to exposing them to unpredictable and unsustainable risks during long-term space flight missions.

## Figures and Tables

**Table 1:** Research on thyroid cells under the effects of altered gravity (green: normal cells, yellow: cancer cells).

Cells	Exposure Device	Dur.	Analyses and Most Important Findings		Ref.
**HUMAN CELLS**					
Primary thyrocytes	**s-µ*g*** RCCS	14 d	Morphology: spheroid formation Protein content: thyroglobulin↑, KGF↑		[51]
HTU-5	**s-µ*g*** RPM	1 d 2 d 3 d	Immunofluorescence microscopy: cytokeratin filaments thickened and shortened, extended from poorly defined organizing centers; vimentin formed a coiled aggregate closely associated with the nucleus		[14]
		3 d	STRING network analysis Mass spectrometry: high quantities of glycolytic enzymes and marginal quantities of citric acid cycle enzymes		[15,52]
Nthy-3-1-ori	**s-µ*g*** RPM	7 d 14 d	Morphology: spheroid formation Gene expression of genes involved in cytoskeleton forming (*ACTB*↑, *TUBB*↑, *PFN1*↑), growth (*OPN*↗, *CPNE1*↑, *TGM2*↑→, *NGAL*↑, *COL1A1*↑, *VEGF*↓↑) and signaling (*IL6*↑, *IL8*↑, *IL17*↗, *PBK*↑, *CASP9*↑, *ERK1/2*↑) Protein content (MAP profiling): IL-6↑, BDNF↑, MMP-3↑, VEGF↓		[16]
	**1.8**× ***g*** SAHC	2 h	Gene expression of genes involved in apoptosis (*TNFA*↓), extracellular matrix (*VCAM*↑), growth (*OPN*↓), cytoskeleton (*ABL2*↑, *ACTB*↑, *ITGA10*→), signaling (*CTGF*↑).		[21]
	**Vibration** Vibraplex		Gene expression of genes involved in apoptosis (*ANXA2*↓, *TNFA*↓), extracellular matrix (*ADAM19*↓, *ITGB1*↓), cytoskeleton (*ACTB*↓, *VIM*↓), and signaling (*PRKAA1*↓,*PRKCA*↓)		
ML-1	**r-µ*g*** PFC	22 s	Relationship between cytoskeleton and ECM under altered gravity Morphology: F-actin/cytokeratin cytoskeleton altered, no signs of apoptosis or necrosis Microarray: 2430 significantly regulated transcripts Gene expression of genes involved in forming cytoskeleton (*ACTB*↑, *LIMA1*↗), and extracellular matrix (*KER80*↑, *COL4A5*↓, *OPN*↑, *FN*↑). *MTSS1*↓		[20]
	**s-µ*g*** RPM (CN)	12 h	Morphology: spheroid formation Protein content: ECM proteins↑, TGF-β↑ Protein secretion: Tg↓, fT3↓, fT4↓,		[12]
		2 d	Morphology: spheroid formation, signs of apoptosis Protein content: apoptosis: Fas↑, p53↑, Bax↑, Casp3↑		[53]
		1 d 2 d	Morphology: spheroid formation, induced apoptosis Protein content: fT3↓, fT4↓, elevated intermediate filaments, cell adhesion molecules, and extracellular matrix proteins		[13]
		1 d 2 d 3 d	Immunofluorescence microscopy: cytokeratin filaments coalesced and shortened, extended from poorly defined organizing centers; enormous elevation on vimentin filaments Protein content: Talin↑, α-tubulin↑, β-tubulin↑, β1-integrin↑		[14]
		3 d 7 d	Morphology: spheroid formation Protein content: IL-6↑, MCP-1↑, integrin-β1↓ in spheroids		[17]
	**s-µ*g*** RPM	7 d 11 d	Proteome analysis Protein content: glutathione S-transferase P↑, nucleoside diphosphate kinase A↑, heat shock cognate 71 kDa protein↑ Mass spectrometry: 202 different polypeptide chains identified compared to 1*g* controls (glycolytic enzymes, structural proteins, cytoplasmic actin, tubulin, various heat shock proteins), many proteins showed different Mascot scores		[54]
	**1.8*g*** SAHC MuSIC	22 s	Gene expression of genes involved in forming cytoskeleton (*ACTB*→, *LIMA1*↑), and extracellular matrix (*KER80*→, *COL4A5*→). *MTSS1*↑		[20]
		2 h	Gene expression of genes involved in cytoskeleton modulation (*EZR*↑, *RDX*↑, *MSN*→), growth factors (*EGF*→, *CTGF*↑), and signaling (*IL6*↓↑, *IL8*↓↑, *PRKAA1*↘↑, *PKC*→)		[24]
	**Vibration** Vibraplex	22 s	Gene expression of genes involved in forming cytoskeleton (*ACTB*→, *LIMA1*→), and extracellular matrix (*KER80*→, *COL4A5*→). *MTSS1*→		[20]
		2 h	Gene expression of genes involved in cytoskeleton forming (*MYO9B*→, *TUBB*→, *VIM*→) and cytoskeleton modulation (*EZR*→, *RDX*→, *MSN*→), growth factors (*EGF*→, *CTGF*↗), and signaling (*IL6*↓, *IL8*↘, *PRKAA1*↘, *PKC*↘)		[24]
UCLA RO82-W-1	**s-µ*g*** RPM CN	3 d 7 d	Morphology: spheroid formation Protein content: integrin-β1↓ in spheroids		[17]
CGTH W-1	**s-µ*g*** RPM	3 d	Morphology: collagen-chains found Gene expression: *VIM*↓, *TUBB*↓, *ACTB*↓		[55]
			STRING network analysis: Considerable number of candidates for gravi-sensitive proteins detected. Clusters of strongly interacting enzymes involved in carbohydrate metabolism, protein Mass spectrometry: low quantities of glycolytic enzymes and marginal quantities of citrate cycle enzymes, abnormal LDH A-chains		[15,52]
	**Vibration** Vibraplex	2 h	Gene expression of genes involved in cytoskeleton forming (*ACTB*↑, *MYO9B*→, *TUBB*↓, *VIM*→, *ITGB1*↑) and cytoskeleton modulation (*EZR*↗, *RDX*→, *MSN*↑), growth factors (*EGF*↗, *CTGF*↑), and signaling (*IL6*↑, *IL8*→, P*KC*↑)		[24]
FTC-133	**r-µ*g*** Space	10 d	*Shenzhou-8/SIMBOX-mission*		[32,33]
			Morphology: spheroid formation Microarray: 2881 significantly regulated transcripts; genes involved in several biological processes: apoptosis, cytoskeleton, adhesion/extracellular matrix, proliferation, stress response, migration, angiogenesis, signal transduction, regulation of cancer cell proliferation and metastasis Gene expression of genes involved in extracellular matrix (*OPN*↓), growth (*EGF*↑, *CTGF*↑, *VEGFA*↓, *VEGFD*↑), and signaling (*IL8*↓)		
		12 d	*ISS/Cellbox 1-mission*		[56]
			Morphology: no spheroid formation Mass spectrometry: 180 different polypeptide chains identified Protein content: enhanced production of proteins related to the extracellular matrix		
	**r-µ*g*** TEXUS-52	369 s	Life-cell imaging (FLUMIAS) with FTC-133 cells expressing the Lifeact-GFP marker protein for the visualization of F-actin: significant alterations of the cytoskeleton		[19]
	**r-µ*g*** PFC	~3 h	Microarray: 63 significantly regulated transcripts Gene expression during the PFC was often regulated in the opposite direction compared with the RPM or Space		[33]
			Life-cell imaging Gene expression of genes involved in cytoskeleton forming (*EZR*↑) and signaling (*SEPT11*↓)		[19]
	**s-µ*g*** CN	4 h 1 d 3 d	Morphology: spheroid formation Gene expression: *CAV1*↓ and *CTGF*↓ in spheroids	Protein content: decreased cytokine release	[10]
	**s-µ*g*** RPM	4 h 1 d 3 d		Protein content: increased cytokine release	
		1 d	Analysis of MCS formation Morphology: spheroid formation, apoptosis enhanced Microarray: 487 significantly regulated transcripts Protein content: NF-κB p65↑ Gene expression: AD: *IL6*↑, *IL8*↑, *CD44*↑, *OPN*↑, *ERK1/2*↓, *CAV2*↓, *TLN1*↓, *CTGF*↓; MCS: *ERK2*↓, *IL6*↓, *CAV2*↓, *TLN1*↓, *CTGF*↓		[57]
		3 d	Proteomic analysis with focus cytoskeletal and membrane-associated proteins to understand forming of larger MCS by FTC-133 cells FF-IEF/SDS-PAGE/mass spectrometry: integrin α5 chains, myosin-10 and filamin B only found in protein solution of FTC-133 cells → possible role in binding fibronectin Gene expression: *VIM*↑, *TUBB*↑, *ACTB*↓		[55]
		3 d	STRING network analysis: considerable number of candidates for gravi-sensitive proteins detected. Clusters of strongly interacting enzymes involved in carbohydrate metabolism, protein formation, degradation, and cell shaping and proteins regulating cell growth. Mass spectrometry: high quantities of glycolytic enzymes and moderate quantities of citric acid cycle enzymes, abnormal LDH B-chains		[15,52]
	**s-µ*g*** RPM	10 d	Morphology: spheroid formation Microarray: 2881 significantly regulated transcripts Gene expression: of genes involved in extracellular matrix (*OPN*↑), growth (*EGF*↑, *CTGF*↑, *VEGFA*↓, *VEGFD*↑), and signaling (*IL8*↓)		[32,33]
	**s-µ*g*** RPM	7 d 14 d	Morphology: formation of larger and numerous spheroids than normal cells Gene expression of genes involved in cytoskeleton forming (*ACTB*↑, *TUBB*↑, *PFN1*↑), growth (*OPN*↑, *CPNE1*↑, *TGM2*↑, *NGAL*↑, *COL1A1*↓, *VEGF*↓→) and signaling (*IL6*↑, *IL7*↑, *IL8*↑, *IL17*↗, *PBK*→, *CASP9*↑, *ERK1/2*↑, *FLT1*↑→, *FLK1*↑→) Protein content (MAP profiling): IL-6↑, MIP-1α↑, IL-1β↑, IL-1ra↑, IL-12p40↑, IL-12p70↑, IL-15↑, IL-17↑, SCF↑, VEGF↓, NGAL↑		[16]
	**1.8**× ***g*** SAHC	2 h	Life-cell imaging Gene expression of genes involved in cytoskeleton forming (*ACTB*↑, *EZR*↑, *RDX*↑, *MSN*↑) and signaling (*LCP1*↑)		[19]
	**Vibration** Vibraplex	2 h	Life-cell imaging Gene expression of genes involved in cytoskeleton forming (*MSN*↓) and signaling		
**ANIMAL MODELS**					
FRTL-5 (rat)	**s-µ*g*** CN	5-7 d	Activity: less-responsive to TSH stimulation in terms of cAMP		[58]
	**5*g*/9*g*** LSC	1 h	cells functionally respond to the variable gravity force in a dose-dependent manner in terms of cAMP production following TSH-stimulation		[23]
	**r-µ*g*** TEXUS-44	379 s	Morphology: irregular shape, rearrangement of the cell membrane Activity: no response to TSH, shedding of TSH-R in the supernatant Protein content: Bax↑, sphingomyelin-synthase↑		[18]
Thyroid gland (mouse)	**r-µ*g*** Space	91 d	Morphology: increase in average follicle size Protein content: sphingomyelinase↑, sphingomyelin-synthase↑		[29,30]
		3 mo	Morphology: thyroid follicles appeared more organized Protein content: caveolin-1↑, TSH-R↑		[1]
		90 d	Protein content: HBME-1↑, galectin-3↑		[47]
	**2× *g*** centrifuge	90/91 d	Protein content: TSHR↑ , caveolin-1↑, STAT3↓ cholesterol↓ , cAMP→		[28,30]

AD, adherent cells; CN, clinostat; Dur., duration; d, day; h, hour; LSC, low speed centrifuge; MCS, multicellular spheroids; mo, month; MuSIC, multi-sample incubator centrifuge; PFC, parabolic flight campaign; r-µ*g*, real microgravity; RPM, random positioning machine; s-µ*g*, simulated microgravity; s, second; SAHC, short-arm human centrifuge; ↑, upregulation; ↓, downregulation; ↗, slight upregulation; ↘, slight downregulation; →, not regulated.

## Abbreviations

2D	Two-dimensional
3D	Three-dimensional
fT3	Free triiodothyronine
fT4	Free thyroxine
*g*	Gravity, acceleration
MCS	Multicellular spheroids
MIB-1	Ki-67 equivalent antibodies
PKA	Protein kinase A
PKC	Protein kinase C
PPAR	Peroxisome proliferator-activated receptor
RPM	Random Positioning Machine
SM	Sphingomyelin
STAT3	Signal transducer and activator of transcription 3
TSH	Thyroid-stimulating hormone
TSHR	Thyroid-stimulating hormone receptor
µ*g*	Microgravity
